# Correspondence

**Published:** 1872-05

**Authors:** Junius E. Cravens, George A. Mills


### CORRESPONDENCE.

KANSAS CITY, MO.,
April 17, 1872.

DR. TAFT, *Dear Sir:* I have heard of a family in which three old maids, sisters, had one set of "store teeth" between them, wearing them alternately. And I actually knew of a *cobbler* who filled two adjoining proximal cavities with the same piece of amalgam for both. That is what I should call *a double header.*

Many incidents in the above strain are reported,—true and fabricated. However, I have a bona fide case to report:

In February last, a German brought his wife to my office. She took the chair, saying that her teeth did not fit "anymore already." I saw the plate was a temporary one—or at least it mounted plain teeth, and the gum was at quite a distance—receded. I wished to remove the plate, but she brought me up with a sharp "no! that set of teeth was to stay in always. The dentist who put them in said *she must never take them out.*" How long, dear reader, do you suppose she had worn that plate clasped fast in her mouth? In the name of all the glue factories on earth—hold your nose! *Nine Years.*

The plate was of gold, and the teeth soldered on. It was clasped to the left superior canine firmly, and to one of the right superior molars! All the intervening teeth were artificial. I wanted to extract the *teeth and plate too,* with the privilege of keeping them, just in the condition surrounding them in the mouth, but I could not persuade the woman to allow it to be done just then.

I am waiting for that patient still, and shall do the extracting, and construct a plate free of charge, rather than miss getting possession of that plate, to start a museum.

There is no valuable information in this communication, but I wish the gentlemen of the profession to know to what extremes of dirt and nastiness some practitioners will pass.

Without giving his name—that dentist enjoyed a large practice in Jacksonville, Ill. Truly Yours,

JUNIUS E. CRAVENS.

BROOKLYN, N. Y.
April 10, 1872.

DR. TAFT: On page 118 of the March number, of the REGISTER, I have just been reading an article on "Neuralgia," by N. E. Wallace, M. D.

By the title affixed, I conclude his claim is Doctor of Medicine. If that is so, I think it a mistake that he writes for a Dental journal. It is no uncommon event that members of the medical profession often do *both* things, mentioned in the article, *i. e. utterly* fail to diagnose the case, and make the egregious blunder of extracting teeth without a *squirm.*

I do not think it strange that Doctors of Medicine make these *frequent and painful* mistakes. They do not familiarize themselves with the Dental field to any extent, (as a rule) and, I am afraid, that *he* who shows such ignorance, and steps out so *carelessly* into the dental field would not be the trusty one in his own domain. If this Wallace is a so-called dentist, let me say, while he deprecates the noble profession from which he received his M. D., he also belittles another, also *noble*, by degrading it to *tooth howling.*

Now, I am this brother's dear friend, if he is in earnest for the *living truth.* If not, I can only pity. I can tell him a better way than that in which he is traveling.

Such cases as he has instanced (as a rule) can be dealt with more intelligently.

You were able to diagnose the case of Widow M. B. Now, if you had adopted the curative treatment, by the use of the science you should have, (judging from your title) you might have hoped for success. If medical science does not afford a sufficient amount of knowledge to meet such cases, then dental science *does.* And I am ready and eager to prove it, not only by our practice, but by that of many others.

What an appeal to an earnest writer on dental education? "Who is sufficient for these things?"

Brother Wallace, I may be *humble*, but I am earnest. If you are for *light*, I am still with you to the brink, and no fee.

GEORGE A. MILLS.

